# Imported Infectious Diseases in Mobile Populations, Spain

**DOI:** 10.3201/eid1511.090718

**Published:** 2009-11

**Authors:** Begoña Monge-Maillo, B. Carolina Jiménez, José A. Pérez-Molina, Francesca Norman, Miriam Navarro, Ana Pérez-Ayala, Juan M. Herrero, Pilar Zamarrón, Rogelio López-Vélez

**Affiliations:** Ramón y Cajal Hospital, Madrid, Spain

**Keywords:** Infectious disease, Chagas disease, tuberculosis, immigrants, hepatitis, parasites, malaria, filariasis, research

## Abstract

Migration has contributed to the emergence of certain infectious diseases. To determine which infectious diseases were most common among 2 mobile immigrant groups (sub-Saharan Africans and Latin Americans) in Spain, we analyzed health and demographic characteristics of 2,198 immigrants referred to the Tropical Medicine Unit of Ramón y Cajal Hospital over a 20-year period. The most frequent diagnoses were for latent tuberculosis (716 patients [32.6%]), filariasis (421 [19.2%]), hepatropic virus chronic infection (262 [19.2%]), intestinal parasites (242 [11.0%]), and malaria (212 [9.6%]). Health screening of immigrant populations is needed to ensure early diagnosis and treatment of potentially transmissible infections.

## Abstract 2

Health screening of immigrant populations is needed to ensure early diagnosis and treatment.

Migration to the European Union has increased exponentially during the past 2 decades, with 1.9 million new registered immigrants in 2008 alone (*1*). Of these, 700,000 arrived in Spain, currently the main recipient country in Europe. The number of documented immigrants in Spain increased from 0.5 million in 1995 to 5.2 million on January 1, 2008, representing 11.3% of the country’s total population (*2*). Thus, Spain may be representative of the impact of migration on certain emerging infectious diseases.

In mobile populations, characteristics and time of acquisition of infections depend on exposure in the original country, during migration, and in the resettlement environment, leading to considerable heterogeneity in presentation. With a few exceptions (e.g., American trypanosomiasis), most tropical infections present no transmission risk for the host population (*3*). However, other infections that affect immigrants and are not exclusive to tropical areas, such as tuberculosis (TB) and HIV, can be transmitted (*4*). Because infections can be introduced in previously unexposed populations and incidence of preexisting infections highly prevalent in countries of origin may increase, the impact of mobile populations on public health should be addressed.

This article compares the characteristics and relevance of infectious diseases in 2 mobile immigrant groups in Spain: sub-Saharan Africans and Latin Americans. Two aims of the study were to improve awareness among clinicians of emerging infections associated with human mobility and to provide additional information about imported diseases. Appropriate medical management also would be expected to affect public health.

## Methods

### Study Population

The Tropical Medicine Unit (TMU) is a referral center at the infectious diseases department of the Ramón y Cajal Hospital in Madrid, Spain. In parallel with clinical work, we collected data about Latin American and sub-Saharan African immigrants seeking health care at TMU from April 1989 through June 2008 and conducted an epidemiologic and clinical study. We excluded from the study immigrants classified as visiting friends and relatives, patients lost to follow-up, and patients with incomplete tests as of June 2008.

In Spain, basic health coverage is universal, and patients need only to possess a health card. Immigrants can acquire this card regardless of their residency status (legal or illegal). If cultural or linguistic differences make obtaining a health card difficult, immigrants can be referred by nongovernmental organizations (NGOs). Patients are therefore referred from primary caregivers, specialists, or NGOs, or they can seek medical care on their own initiative (because of symptoms or to request a health examination). Most immigrants seen at our unit have migrated for social or economic reasons and are from outside the European Union, primarily Latin America and sub-Saharan Africa.

### Diagnoses

Demographic variables included age, sex, country of origin, health coverage (defined as holding Spain’s national health card), and preconsultation period (defined as months from arrival in Spain to first consultation at TMU). We grouped patients’ primary reasons for seeking medical assistance at TMU into 10 syndromes: dermatologic, febrile, gastrointestinal, respiratory, genitourinary, neurologic, musculoskeletal, hematologic–anemia, hematologic–eosinophilia, and asymptomatic. Each patient could be assigned to >1 of these categories.

Screening for asymptomatic patients comprised blood count, biochemistry, basic urinalysis, HIV serologic analysis, hepatitis B virus (HBV) and HCV serologic analysis, rapid plasma reagin, Mantoux skin test, stool parasites, PCR for malaria in sub-Saharan Africans (since 2005), and Chagas disease serologic analysis (immunofluorescent antibody test, ELISA) and PCR (since 2003) in persons from Latin America. Infectious diseases were diagnosed following standard techniques and grouped into 4 categories.

The first category was tropical infectious diseases, which were infections typically imported from tropical areas into Spain, even though some may be distributed worldwide. Examples include filariasis, malaria, trypanosomiasis*,* cysticercosis, schistosomiasis, and intestinal parasites.

The second category was transmissible infectious diseases, which were infections distributed globally but more prevalent in the countries of origin, with a high risk for transmission in the host country and that comprise a substantial proportion of cases in Spain. Examples include latent and active TB, acute and chronic infections with hepatotropic virus, sexually transmitted infections (STI), HIV infection, and leprosy.

The third category was common infectious diseases, which were infections distributed worldwide and prevalent in tropical and nontropical areas but which are not the focus of this study. Examples include respiratory tract infections, gastrointestinal bacterial infections, and urinary tract infections.

The fourth category was infrequent infectious diseases, which were infections with <10 cases per diagnosis. Examples include toxoplasmosis, amebic liver abcesses, and leishmaniases.

### Statistical Analysis

We calculated frequency rates for reason for referral and infectious diagnoses for Latin Americans and sub-Saharan Africans. Qualitative variables were compared using the χ^2^ test, the Fisher exact test, or the χ^2^ test for linear trends when necessary. For quantitative variables, the Student t test for nonpaired variables or the Mann-Whitney U test were used. Significance was designated at p<0.05. All tests were performed with the SPSS 15 software for Windows (SPSS Inc., Chicago, IL, USA).

## Results

Demographic characteristics of the study population are shown in Table 1. The most frequent countries of origin for sub-Saharan African immigrants were Equatorial Guinea (35.7%), Nigeria (13.3%), Senegal (7.4%), and Cameroon (6.2%); for Latin Americans, they were Ecuador (34.9%), Bolivia (27.8%), Peru (11.2%), and Colombia (8.8%).

**Table 1:** Demographic characteristics of immigrant population seeking care at the Tropical Medicine Unit, Ramón y Cajal Hospital, Madrid, Spain, 1989–2008*

Characteristic	Total	Sub-Saharan Africans	Latin Americans	p value
Study participants, no. (%)	2,198 (100)	1,564 (71.2)	634 (28.8)	
Male sex, no. (%)	1,303 (59.3)	882 (56.4)	421 (66.4)	<0.001
Median age, y (IQR)	29 (22–36)	28 (22–35)	32 (24–40)	
Median preconsultation period, mo†	7	5	19	<0.001
Health coverage,‡ no. (%)	739 (33.6)	348 (22.3)	391 (61.7)	<0.001

*IQR, interquartile range (25th–75th percentile). †Defined as months elapsed from arrival to Spain to first consultation at the Tropical Medicine Unit. ‡Defined as holding Spain’s national health card.

During the 20-year study period, the number of patients seen increased significantly (β = 10, p<0.001), as did the proportion of men and the proportion of Latin American immigrants. Three other variables (preconsultation period, age, and health coverage) did not differ significantly. Table 2 shows frequencies for each reason for seeking medical assistance according to areas of origin and grouped in 10 syndromes.

**Table 2:** Immigrants’ reasons for seeking medical assistance, by area of origin, Tropical Medicine Unit, Ramón y Cajal Hospital, Madrid, Spain, 1989–2008*

Syndrome	Total population, no. (%), N = 2,198	Sub-Saharan Africans, no. (%), n = 1,564	Latin Americans, no. (%), n = 634	p value	
Hematologic–eosinophilia	570 (26)	435 (28)	135 (21.3)	0.002	
Dermatologic	544 (24.7)	477 (30.5)	67 (10.6)	0.001	
Fever	451 (20.5)	351 (22.4)	100 (15.8)	0.001	
Asymptomatic	396 (18)	268 (17.1)	128 (20.2)	0.09	
Gastrointestinal	363 (16.5)	269 (17.2)	94 (14.8)	0.608
Respiratory	314 (14.3)	209 (13.4)	105 (16.6)	0.006	
Hematologic–anemia	283 (12.9)	230 (14.7)	53 (8.4)	0.001	
Genitourinary	234 (10.6)	198 (12.7)	36 (5.7)	0.001	
Neurologic	219 (10)	144 (9.2)	75 (11.8)	0.03	
Musculoskeletal	169 (7.7)	141 (9)	28 (4.4)	0.001	

*Because each patient could have >1 main reason for seeking medical assistance, the number of cases can be higher than the number of patients. Percentages were calculated as number of cases divided by number of patients in each group (total population, sub-Sahara African immigrants, or Latin American immigrants).

For 2,088 (95.0%) of the 2,198 patients a final diagnosis of infectious or noninfectious diseases was made; 110 (5.3%) had no evidence of illness. Of patients who received >1 diagnosis. 1,377 (65.9%) had multiple diagnoses (>10/patient): 34.1%, 28.9%, 17.8%, 10.1%, and 4.5% had 1, 2, 3, 4, and 5 diagnoses, respectively. Among those classified as asymptomatic at first consultation, 87.8% had evidence of chronic disease (e.g., hypertension, diabetes, hypercholesterolemia, iron deficiency anemia, hemoglobinopathies, thyroid disease) or various infections (Tables 2, 3, 4).

**Table 3:** Disease diagnoses in immigrants, by area of origin, Tropical Medicine Unit, Ramón y Cajal Hospital, Madrid, Spain, 1989–2008*

Diagnostic category and disease	Total population, no. (%), N = 2,198	Sub-Saharan Africans, no. (%), n = 1,564	Latin Americans, no. (%), n = 634	p value
Tropical infectious diseases				
Filariasis	421 (19.2)	418 (26.7)	3 (0.4)	0.001
Intestinal parasites	242 (11.0)	162 (10.4)	80 (12.6)	0.15
Malaria	212 (9.6)	199 (12.7)	13 (2.1)	0.001
Chagas disease	101 (4.5)	0	101 (15.9)	
Schistosomiasis	39 (1.8)	38 (2.4)	1 (0.2)	0.001
Cysticercosis	31 (1.4)	3 (0.2)	28 (4.4 )	0.001
Transmissible infectious diseases				
Latent tuberculosis	716 (32.6)	596 (61.2)	120 (18.9)	0.001
Active tuberculosis	107 (4.8)	52 (3.3)	55 (8.7)	0.001
Hepatotropic virus, acute infection†	31 (1.4)	27 (1.7)	4 (0.6)	0.075
Hepatotropic virus, chronic infection‡	262 (11.9)	257 (16.4)	10 (1.6)	0.001
Sexually transmitted infections§	107 (4.9)	92 (5.9)	15 (2.4)	0.002
HIV infection	97 (4.4)	82 (5.2)	15 (2.4)	0.005
Leprosy	8 (0.4)	3 (0.2)	5 (0.8)	0.02
Common infectious diseases				
Respiratory infections	61 (2.8)	36 (2.3)	25 (3.9)	0.013
Gastrointestinal bacterial infections	92 (4.2)	69 (4.4)	23 (3.6)	0.705
Urinary infections	69 (3.1)	45 (2.9)	24 (3.8)	0.135
Skin infections	80 (3.6)	71 (4.5)	9 (1.4)	0.001
Infrequent infections	36 (1.7 )	20 (1.3)	16 (2.5)	0.025
Noninfectious diseases	596 (27.1)	430 (27.5)	166 (26.2)	0.978

*Because each patient could have >1 diagnosis, the number of cases can be higher than the number of patients. Percentages have been calculated as number of cases divided by number of patients in each group (total population, sub-Sahara African immigrants, or, Latin American immigrants). †Acute infections with hepatotropic virus caused by hepatitis A virus, hepatitis B virus, hepatitis E virus, cytomegalovirus, and Epstein-Barr virus ‡Chronic infections with hepatotropic virus were caused by hepatitis B virus, hepatitis C virus, and hepatitis D virus. §Sexually transmitted infections comprised syphilis, bacterial vaginosis, trichomoniasis, gonococcal urethritis, *Chlamydia trachomatis*, and genital herpes virus.

**Table 4:** Infectious diseases diagnoses in asymptomatic patients, Tropical Medicine Unit, Ramón y Cajal Hospital, Madrid, Spain, 1989–2008

Diagnostic category and disease	Asymptomatic cases, no. (%), n = 396
Tropical infectious diseases	
Filariasis	36 (9.1)
Intestinal parasites	35 (8.8)
Malaria	15 (3.8)
Chagas disease	43 (10.9)
Schistosomiasis	5 (1.3)
Cysticercosis	0
Transmissible infectious diseases	
Latent tuberculosis	160 (40.4)
Active tuberculosis	3 (0.7)
Hepatotropic virus, acute infection*	2 (0.5)
Hepatotropic virus, chronic infection†	40 (10.1)
Sexually transmitted infections‡	10 (2.5)
HIV infection	19 (4.8)
Leprosy	0
Common infectious diseases	
Respiratory tract infections	0
Gastrointestinal bacterial infections	10 (2.5)
Urinary tract infections	9 (2.3)
Skin infections	9 (2.3)
Infrequent infections	8 (2.0)

*Acute infections with hepatotropic virus caused by hepatitis A virus, hepatitis B virus, hepatitis E virus, cytomegalovirus, and Epstein-Barr virus. †Chronic infections with hepatotropic virus were caused by hepatitis B virus, hepatitis C virus, and hepatitis D virus. ‡Sexually transmitted infections comprised syphilis, bacterial vaginosis, trichomoniasis, gonococcal urethritis, *Chlamydia trachomatis*, and genital herpes virus.

### Tropical Infectious Diseases

#### Filariasis

We found 421 filariasis cases, of which most occurred in sub-Saharan Africans. Of these, 258 were *Onchocerca volvulus* infections, 124 were *Mansonella perstans* infections, 29 were *Loa loa* infections, and 7 were *M*. *streptocerca* infections. Three cases in Latin American patients were caused by *O. volvulus* infections.

#### Intestinal Parasites

Intestinal parasites were diagnosed in 242 patients, mostly from sub-Saharan Africa. The most frequent parasites identified were *Ascaris lumbricoides* (35.5%) and *Giardia intestinalis* (28.5%).

#### Malaria

Malaria, diagnosed in 212 patients, occurred significantly more often in sub-Saharan Africans than in Latin Americans. The median preconsultation period was 2 months. Fifteen (7.1%) patients were asymptomatic when their malaria was diagnosed. Among the 199 sub-Saharan African malaria patients, 125 were infected with *Plasmodium falciparum*, 13 with *P. malariae*, 10 with *P. ovale*, 4 with *P. vivax*, 8 with mixed infections (5 with *P. falciparum* and *P. malariae*, 2 with *P. falciparum* and *P. ovale*, and 1 with *P. malariae* and *P. ovale*), and 39 with *Plasmodium* sp. where specific species could not be determined, generally because the diagnosis was made when PCR was not available. Malaria was diagnosed in 13 Latin American patients; 10 had *P. vivax* infections and 3 had *P. falciparum* infections*.*

#### Chagas Disease

Of the 101 cases of Chagas disease diagnosed, all were in Latin American patients, with 95.0% from Bolivia, and most cases (71.3%) occurred in men. Of Chagas disease cases, 42.6% were asymptomatic and were detected after routine screening. Organ involvement was found in 20.7% of patients; 17.5% had cardiac disease, 2.0% had gastrointestinal involvement, and 1.2% had both.

### Transmissible Infectious Diseases

#### Tuberculosis

Latent TB infection (716 cases) occurred significantly more often in sub-Saharan African than Latin American patients. Active TB (107 cases) occurred more often among Latin Americans; the highest proportions of patients were from Ecuador (21.4%) and Peru (17.5%). The median preconsultation period was 12 months. Of active TB cases, 75 (70.1%) were pulmonary TB and 32 (30%) were extrapulmonary TB. Co-infection with HIV and active TB was detected in 12 (11.2%) patients, mostly from sub-Saharan Africa (75.0%).

#### Hepatitis

A total of 262 patients had chronic infection with hepatitis viruses. The prevalence was higher in sub-Saharan Africans. The most frequently identified chronic hepatitis virus was HBV (60.7%). The prevalence of hepatitis B surface antigen in sub-Saharan African patients was 9.8%. The prevalence of coinfection with HBV and HCV was 1.6%, and 0.9% with HBV and hepatitis D virus. Acute hepatitis infection was diagnosed infrequently: 41 (1.4%) cases, with no significant differences between the 2 groups. In addition, 797 patients had evidence of past HBV infection, of whom 277 (34.7%) had antibody to virus core or virus core protein; 95.7% were from sub-Saharan Africa.

#### Sexually Transmitted Infections and HIV

STIs were found in 112 patients, mainly from sub-Saharan Africa. Sixty-seven patients had latent syphilis, 17 had bacterial vaginosis, 12 had trichomoniasis, 9 had genital herpes, 4 had *Chlamydia trachomatis* infections, and 3 had gonococcal urethritis. Among patients in whom syphilis was diagnosed, 59 (88.0%) were from sub-Saharan Africa and 8 (11.9%) were from Latin America, 36 (53.7%) were men, and 10 (15%) were co-infected with HIV. Six were asymptomatic when they sought care at TMU. HIV was diagnosed in 97 patients; 19 were asymptomatic and 82 (84.5%) were from sub-Saharan Africa.

### Infrequent Infections

Nine patients had toxoplasmosis, 8 had amebic liver abscesses; 5 had rickettsiosis; 4 had *Fasciola hepatica* infections; 2 each had cutaneous leishmaniasis, mucocutaneous leishmaniasis, and brucellosis; and 1 each had visceral leishmaniasis, hydatid disease, borreliosis, and human T-lymphotrophic virus type 1 infection. These infrequent infections occurred proportionally more frequently among Latin American than among sub-Saharan African patients.

## Discussion

The large size of the population analyzed and the long time period (≈20 years) add strength to the study. Because one third of the study population were considered illegal aliens and lacked health insurance, our analysis was able to provide valuable information about infections affecting a group that is frequently not adequately represented in published studies because of the usual restrictions on access to health services. Nonetheless, this study is subject to several limitations.

In Spain, most immigrants come from a European Union member state (Romania, 14% of all immigrants in Spain), followed by northern Africa (Morocco, 12%) and Latin America (Ecuador, 8%) (*2*). Extrapolation of the results of this study to the global immigrant population may therefore be limited. However, these mobile groups are important because of the spectrum of imported diseases represented. This applies both to tropical diseases and transmissible infections (*5*).

Comparison based on geographic distribution may exclude other important aspects involved in the development of certain infectious diseases: socioeconomic status of the country, public health infrastructure, rural or urban origin, or reason for migration. However, characteristics of most immigrants seen at TMU were similar: most came from tropical, underdeveloped areas of Latin America and sub-Saharan Africa, and most migrated for economic reasons.

During the 20 years of data collection, diagnostic methods and screening procedures changed, thus influencing results over time. Depending on how the patients were referred to our clinic the frequency of the various diseases reported cannot be interpreted as prevalence rates in the 2 migrant populations considered. Because most individuals were referred for investigation of symptoms or diseases, the frequency observed can be expected to be higher than in the overall migrant population.

Demographic variables of age and sex are in accordance with national data on immigrant populations (*2*) and are similar to other series (*6*,*7*), except for the larger proportion of males in the Latin American group. The significant difference in the preconsultation periods of the 2 groups might be explained by the larger proportion of Latin Americans who held health cards and thus were seen initially by general practitioners. Because most sub-Saharan African immigrants were undocumented and referred from NGOs, TMU was their first contact with the public health system. Nevertheless, the proportion of Latin Americans seen at TMU as a result of active health promotion campaigns and screening for Chagas disease increased.

Consistent with other series (*8*), the most frequent reasons for seeking medical assistance at TMU were hematologic–eosinophilia, dermatologic syndrome, and fever. All syndromes occurred significantly more often in sub-Saharan Africans, except for respiratory and neurologic. Geographic variation in disease distribution would explain certain differences. The increased frequency of dermatologic syndrome and eosinophilia in Africans could be due to the greater incidence of filariasis; anemia could result from nutritional deficiencies and hemoglobinopathies; and fever could be due to malaria. For Latin Americans, increased prevalence of neurocysticercosis and TB might explain the increased frequency of neurologic and respiratory syndromes.

The most common tropical disease was filariasis, with onchocerciasis the most frequent filarial infection, affecting sub-Saharans significantly more often than Latin Americans. More than 99% of symptomatic onchocerciasis occurs in western sub-Saharan Africa, where most of the patients in the study originated. The number of cases diagnosed at TMU, however, has progressively decreased since 2000. Screening for filariasis by microfilaremia detection and skin snips should probably be considered in patients presenting pruritus or eosinophilia (*9*).

In Europe, as in Spain, immigrants account for approximately 50% of malaria cases (*10*). In our series, as in other national (*11*) and international (*12*) studies, most malaria patients were from sub-Saharan Africa, and the most commonly isolated species was *P. falciparum*. However, *P. vivax* was the principal species diagnosed in Latin Americans. A considerable number of patients were asymptomatic at diagnosis, probably because of partial immunity developed during residence in areas to which malaria is endemic (*13*) and the implementation of PCR for *Plasmodium* spp. as a screening test in sub-Saharan Africans, which enables detection of very low parasitemia. Malaria screening should be considered in newly arrived immigrants from areas to which it is endemic, regardless of clinical presentation (*14*,*15*); in specialized referral centers, such as TMU, PCR with its higher sensitivity and specificity should be available.

Migration is changing the geographic distribution of Chagas disease, which until recently has been limited to Latin America. During the 1970s, the United States was the leading recipient of Latin American migrants but Europe (especially Spain) is now a main recipient (*16*). A recent study in Spain estimated the number of immigrants potentially infected with *Trypanosoma cruzi* could range from 37,000 to 122,000 (*17*). Alternate, nonvectorial, routes of transmission (vertical or through blood transfusion or organ transplantation) and reactivation episodes in inmunosuppressed persons make Chagas disease an emerging and potentially transmissible disease in the autochthonous population, and thus an important public health concern (*18*). Furthermore, severe cardiac disease could develop in infected persons; a broader knowledge of this aspect of the disease by clinicians is necessary. In our series, prevalence reached nearly 16% among Latin Americans. Bolivian patients accounted for 95% of cases, reflecting its high endemicity (*19*). Electrocardiographic and echocardiographic disorders were found in 15.9% and 7.3% of patients, respectively. These results suggest that all immigrants from areas to which it is endemic should be screened.

In the literature, the prevalence of intestinal parasites in immigrants ranges from 11% to 67%, depending on the origin and type of immigrant, with higher frequencies in recently arrived refugees and sub-Saharan Africans (7,*20*,*21*). However, we found a low prevalence (11%). The impact of intestinal parasitism is low because a considerable proportion of affected persons are asymptomatic, and illness is generally low, with certain parasites (e.g., *Strongyloides* *stercoralis*) (*22*). In our study, 11.5% of patients had no symptoms. These findings question the usefulness of screening the migrant population from areas to which it is endemic for intestinal parasitism. Some authors propose empiric treatment with albendazole (*23*), effective against *S.* *stercoralis* and *G. intestinalis* (*24*), as the most cost-effective measure. Others argue against this measure for reasons such as potential side effects, possible incorrect treatment of certain pathogens (e.g., *Entamoeba histolytica*), and the inherent risks of treating a patient with neurocysticercosis. Although further studies are necessary to assess advantages and risks of empiric treatment over screening (*15*) as a public health measure, an alternative approach could be selective screening of high-risk groups, such as pregnant women, children, and inmunosuppressed patients.

TB is an example of an infectious disease that has emerged with the increase in mobile populations (*25*). Prevalence of active TB among immigrants is higher than in the host population. We found active TB more often in Latin Americans than in sub-Saharan Africans, similar to other national studies (*26*). That TB prevalence rates/100,000 population are as high as 195 and 266 in Ecuador and Bolivia, respectively (World Health Organization data, 2006, http://apps.who.int/globalatlas/dataQuery/default.asp), the most common countries of origin of our Latin American group, might explain this result. Median interval from arrival in Spain to presentation with TB was 2 years, which is consistent with previous studies showing a higher risk for TB during the first 2–5 years of residence in the host country and where activation of latent TB infection seems to be a common cause of TB among immigrants (*27*). The rate of latent TB (32.6%) in our study was consistent with other series (*7*). Latent TB occurred more often in sub-Saharan Africans than in Latin Americans.

Public heath interventions for control of TB in the immigrant population are intensely debated. Many authors consider that interrupting the ongoing community TB transmission through detection and treatment of active TB and contact investigation is the best cost-effective strategy (*28*). However, current guidelines in certain countries recommend screening for latent TB in immigrants, especially those who entered the host country within the previous 5 years (*29*,*30*). Screening policies for latent TB face several difficulties, and no consensus has been reached, the best screening method is under discussion (chest radiographs, intradermal tuberculin test [IDT]), IDT interpretation is ambiguous (3 cutoff points may be considered), the false-positive rate is high because of BCG (Mycobacterium bovis BCG) vaccination, and ensuring high rates of treatment completion for latent TB in the migrant population has proven difficult (*31*). Nevertheless, with the implementation of new screening techniques (interferon gamma release assay) that are less affected by BCG vaccination (*32*) and use of culturally adapted programs to improve adherence to treatment, screening for latent TB followed by appropriate treatment could be a successful strategy for global TB control in Western countries. Some studies support a better, cost-effective approach to TB using screening (*33*).

Chronic HBV infection is a major health problem in sub-Saharan Africa and Asia, where prevalence is >8% (*34*). The mortality rate from chronic infection is ≈25% because of complications (liver cirrhosis and hepatocellular carcinoma). The 9.8% prevalence in our study was similar to others (*7*,*35*). In developing countries, many infections are acquired during the perinatal period, and patients develop long-term complications in early adulthood, whereas in developed countries, infection and complications occur later in life. Screening for HBV in sub-Sahara African immigrants would help detect chronic infections in young adults, allowing early treatment and monitoring for complications. This could also prompt contact screening, vaccination, and education as preventive measures (*15*). Finally, when an isolated core antibody pattern is detected, a relatively common serologic result in the sub-Saharan African population, PCR assessment for occult chronic infection for HBV DNA may be required, especially in HIV-positive patients (*35*).

STIs other than HIV and viral hepatitis were diagnosed in 4.8% of the total population studied. Most STIs were due to latent syphilis (60%), particularly in the Africans. This trend toward more bacterial STIs in certain groups, such as immigrants, is consistent with observations throughout Spain and Europe (*36*). Most STIs, particularly syphilis, can be easily misdiagnosed because they can run a mild or asymptomatic course, but complications and sequelae can be severe. Moreover, syphilis can be easily transmitted from mother to child. Intervention programs for immigrants, particularly sub-Saharan Africans, with active screening and treatment for syphilis could reduce the prevalence and transmission of this infection in the community.

In December 2007, the Joint United Nations Programme on HIV/AIDS (*37*) stated an estimated 33.2 million persons were living with HIV infection worldwide, with most (22.5 million) living in sub-Saharan Africa. In our series, HIV infection also occurred significantly more often among sub-Saharan Africans than among Latin Americans. Throughout western Europe, HIV infection increasingly, and disproportionately, affects immigrants from countries in which the prevalence of HIV/AIDS is high and, in most countries, accounts for the majority of heterosexually acquired HIV infections diagnosed in recent years (*38*). In some series, HIV is diagnosed in immigrants and refugees at a later stage of infection, with lower CD4 cell counts, a larger proportion of females, and with different HIV subtypes (*39*). This highlights the need for screening immigrants, particularly sub-Saharans, for HIV to enable early diagnosis and treatment as well as prevention and education. A larger social and cultural program support is necessary to ensure adequate treatment as well as access to the health care system.

Increased population mobility has led to the disappearance of existing barriers for the spread of certain diseases. Characteristics of mobile populations are becoming increasingly heterogeneous (*4*). Epidemiologic studies in mobile groups help determine factors possibly associated with greater risk for certain pathologies and identify which of the latter may influence health to a greater extent. Our study may improve knowledge about pathology in 2 important mobile populations in Spain, enabling early diagnosis and treatment of potentially transmissible infections (TB), education and prevention programs (STIs, HIV) and catching up on vaccination (*40*), with ensuing positive public health repercussions. Future research may focus on development of diagnostic protocols for imported diseases on the basis of epidemiologic findings.
